# Diffusion Transformer-based Universal Dose Denoising for Pencil Beam Scanning Proton Therapy

**Published:** 2025-06-04

**Authors:** Yuzhen Ding, Jason Holmes, Hongying Feng, Martin Bues, Lisa A. McGee, Jean-Claude M. Rwigema, Nathan Y. Yu, Terence S. Sio, Sameer R. Keole, William W. Wong, Steven E. Schild, Jonathan B. Ashman, Sujay A. Vora, Daniel J. Ma, Samir H. Patel, Wei Liu

**Affiliations:** 1 Department of Radiation Oncology, Mayo Clinic, Phoenix, AZ 85054, USA; 2 College of Mechanical and Power Engineering, China Three Gorges University, Yichang, Hubei 443002, China; 3 Department of Radiation Oncology, Guangzhou Concord Cancer Center, Guangzhou, Guangdong, 510555, China; 4 Department of Radiation Oncology, Mayo Clinic, Rochester, MN 55905, USA

## Abstract

**Purpose:**

Intensity-modulated proton therapy (IMPT) is an advanced treatment modality for head and neck (H&N) cancer patients, offering precise tumor dose coverage while sparing surrounding organs at risk (OARs). However, IMPT is highly sensitive to inter-fractional anatomical changes, necessitating periodic adjustments through online adaptive radiation therapy (oART). But a significant bottleneck in the current oART workflow is the need for fast and accurate dose calculation using Monte Carlo (MC) simulations for plan quality assessment and re-optimization. Reducing the number of particles in MC-based simulations can accelerate dose calculation but at the cost of reduced accuracy. To address this, denoising noisy dose maps generated by low statistics MC simulations has been proposed as a method to rapidly and accurately generate high-accuracy dose maps.

**Methods:**

A diffusion transformer-based dose denoising framework was developed. IMPT treatment plans and 3D CT images from 80 H&N cancer patients were used to construct the training dataset by generating noisy dose maps and their corresponding high statistics dose maps using an open-source MC software, MCsquare, with a computation time of approximately 1 minutes and 10 minutes per plan, respectively. Each data sample was standardized into uniform chunks with zero-padding. Then, normalization and non-linear mapping were applied to the data samples to transform them toward a quasi-Gaussian distribution. The treatment plans and 3D CT images from another independent 10 H&N cancer patients, 10 prostate cancer patients, 10 lung cancer patients, and 10 breast cancer patients were used as the testing dataset, following the same preprocessing protocol as the training dataset. The proposed model was trained with noisy dose maps and 3D CT images as input and high statistics dose maps as the ground truth. The training was constrained by mean square error (MSE) loss, a residual loss that focused on reducing the difference between the predicted and ground truth dose maps and a regional mean absolute error (MAE) loss that specifically targeted voxels with the top 10% and bottom 10% dose values. Performance was evaluated using MAE. 3D Gamma passing rates and dose volume histogram (DVH) indices were calculated to assess differences between the predicted and ground-truth dose maps.

**Results:**

The proposed framework achieved MAE of 0.195 ± 0.112 Gy[RBE], 0.120 ± .054 Gy[RBE], 0 . 172 ± .096 Gy[RBE], and 0.376 ± 0.375 Gy[RBE] for H&N, lung, breast and prostate testing cases, respectively. The 3D gamma passing rate consistently exceeded 92% in the whole body using a 3%/2 mm criterion across all disease sites. DVH indices calculated from the ground truth and predicted dose distributions showed excellent agreement for both clinical target volumes (CTVs) and OARs.

**Conclusion:**

A diffusion transformer-based denoising framework was successfully developed. Although the denoising model was trained using only H&N data, it can accurately and robustly denoise noisy dose maps across different disease sites.

## Introduction:

Intensity modulated proton therapy (IMPT), an advanced form of radiotherapy, employs charged protons instead of conventional X-rays (photons) to target tumors. A key advantage of proton therapy lies in the Bragg Peak phenomenon, which allows protons to deposit the majority of their energy at a well-defined depth in tissue. This characteristic results in minimal exit dose, thereby reducing radiation exposure to normal tissues beyond the tumor and improving dose conformity, making it particularly beneficial for pediatric cancers, tumors near critical organs, and re-irradiation cases^1–7^. Thus, it is a preferred treatment modality for head and neck (H&N) cancer patients due to the complexity and proximity of numerous organs at risk (OARs), such as spinal cord, brainstem and salivary glands, bones, in addition to the swallowing and speech apparatuses, adjacent to the targets. This dosimetric advantage reduces the risk of severe toxicities, including xerostomia, dysphagia, and osteoradionecrosis, thereby improving patient quality of life and treatment outcomes.

Nevertheless, a key challenge in IMPT is its inherent sensitivity to range and setup uncertainties^8–10, 11 , 12–18^. These uncertainties can lead to significant degradation of dose distribution for a proton therapy treatment plan^19–28^. Additionally, it lacks robustness to inter-fractional anatomical changes, which can occur due to patient weight loss, tumor shrinkage or variations in positioning. These anatomical changes can significantly alter the water equivalent thickness of the proton beam path, potentially leading to underdosing of the tumor or overdosing of normal structures. To mitigate these effects, online adaptive radiation therapy (oART)^29, 30^ has emerged as a solution, allowing adaptation of a treatment plan to the patient’s current anatomy.

Yet, a critical bottleneck in the oART workflow is the need for fast and accurate dose calculation, essential for practical online treatment plan adaptation. Monte Carlo (MC) simulations^31–34^ are considered to be the gold standard for proton dose calculation due to their high accuracy in modeling proton interactions with tissue. However, it is computationally expensive and labor intensive, requiring minutes to hours per plan, making real-time plan adaptation infeasible. Another commonly used method is pencil beam algorithms (PBA)^35–38^, which are calculated faster than MC methods. But PBA relies on simplified dose deposition models and is less accurate than MC-based methods, especially for heterogeneous tissues (e.g., near lung-tumor interfaces, paranasal sinus/soft tissue interfaces or metal implants). Thus, both approaches are not ideal for time-sensitive oART workflows in proton therapy.

To address this challenge, modern advances in artificial intelligence (AI) and deep learning, especially their adoption in radiotherapy^39, 40^ have led to the development of data-driven dose prediction and denoising models. These models leverage large datasets of precomputed MC simulations to train neural networks that estimate dose distributions with high accuracy compared to traditional first principle of physics-based approaches while significantly reducing computation time. In 2021, Ahn et al.^41^ developed a patient-specific dose prediction model for left-sided breast cancer using a UNet-based neural network. The model’s performance was compared with conventional knowledge-based planning, showing enhanced efficiency and quality in treatment planning. However, the experiments were performed on a dataset consisting of 60 patients (50 for training and 10 for testing) with left-sided breast cancer, all collected from a single institution. The limited sample size from one single institution makes the resulting model less representative of broader patient populations, potentially leading to poor performance on patients from external institutions. More recently, DoseNet^42^, incorporated radiotherapy background knowledge into deep neural network dose prediction. The study took advantage of the distribution characteristics of high-energy radiation in tissues, aiding prediction of conformal doses within targets. Unfortunately, similar to other studies, the model was trained and tested on limited data within one disease site, lacking the generalizability to other disease sites.

Current deep learning-based models are typically disease-site specific. Therefore, numerous models are needed for different disease sites and/or sub disease sites, which are time-consuming and impractical for routine clinical practices. In addition, the training data are relatively consistent with clear and regular structure contours, which could lead to less robust models and potential model overfit. Few studies have been targeted for highly inhomogeneous disease sites such as H&N. Recently, a 3D dose prediction model DeepDoseNet^43^ was proposed to combine ResNet and Dilated DenseNet architectures. The model was trained and validated using H&N cancer datasets from the 2020 AAPM OpenKBP challenge, demonstrating significant improvements in prediction accuracy. However, the model was disease site–specific and tested only on a well-structured, publicly available dataset. Its applicability to other disease sites is unknown, limiting broader clinical utility. In 2022, Gronberg et al.^44^ developed a 3D Dense Dilated UNet model to predict dose distributions for H&N cancer patients. The model utilized CT images, target prescriptions, and contours of targets and OARs. The predicted dose distributions were comparable to knowledge-based planning (KBP) plans, and the model effectively identified suboptimal plans, suggesting its potential for automated, individualized quality assurance in clinical settings. Another work used a cascade transformer-based model^45^ that combined an encoder-decoder network for OAR segmentation with a pyramid architecture for dose distribution prediction for H&N. Evaluated on both in-house and public datasets, the model outperformed existing baselines, particularly in regions with low dose values. Nonetheless, the model performance for other disease sites remained unreported.

Other than the low generalizability of prior work, existing approaches require CT images, associated structure contours of both clinical target volumes (CTV) and OARs, planning dose, and other customized information to train the networks. Additionally, the data must have uniform size and spacing, which significantly limits the availability of usable datasets and poses an overfitting risk to the networks. Moreover, such trained networks often struggle to adapt to domain-shifted data, including variations from different disease sites, prescription doses, and CT resolutions — factors that are frequently encountered in real-world clinical practice. It is more practical to have a single model trained with data from one disease site without any strictly required input data and that the trained model can be applied to various disease sites without re-training or fine-tuning for routine use.

Thus, herein, we propose a novel dose denoising framework with the diffusion transformer (DiT) as the backbone, aiming for dose denoising, especially for the H&N disease site with limited and loosely constrained input data. We further investigate whether the developed model is robust enough and thus, generalizable to other disease sites without any fine-tuning or re-training. To better align with clinical practice, no constraints were imposed on sub-disease categories, treatment plan configurations, CT image resolutions, or any other patient-specific variables. We aim to develop a robust, generalizable model that advances dose denoising modelling for real-world clinical implementation. The framework was trained and evaluated using data from 90 H&N cancer patients and subsequently tested on an additional 30 patients with lung, breast, and prostate cancers. The proposed method successfully denoised the noisy dose maps with minimal requirements of model input data and excellent performance in other disease sites without fine tuning and re-training.

## Methods and Materials

### Data Collection and Processing

Following institutional review board approval, we collected treatment plans and CT images from 90 patients with H&N cancer who received IMPT at our clinic. We then used an open-source MC software, MCsquare^34^, to recalculate the plan dose to get two dose maps for each patient: (1) a noisy, low statistics dose map using a low number of particles and (2) a clean, high statistics dose map using a much larger number of particles. No further selection criteria to make training data more consistent, such as specific sub disease sites, age, gender, treatment history etc., were enforced to ensure variety in the training data. Therefore, the associated CT images and dose distributions inherently varied significantly across patients, posing a challenge for universal denoising models.

We then developed the following pipeline to standardize input data while preserving patient data variation. The steps include: 1) The noisy dose maps and corresponding high statistics dose maps (the ground truth), regardless of their original sizes, were first vectorized to a 1D vector. 2) The flattened dose maps were divided into non-overlap chunks of size 1×4096 with zero-padding and reshaped to 4×32 ×32 to accommodate the network inputs size. Chunks containing zero-dose value were discarded. 3) The noisy doses were smoothed with Gaussian noise N(0;I) to remove outliers and high frequency noise. 4) All the chunks were augmented by normalization to range 0–1 with respect to the maximum physical dose (Gy[RBE]), then adding 1 to each voxel and applying the logarithm function to amplify variations non-linearly, especially for voxels with small dose value in the distal edge of the penumbra regions. 5) The CT images were flattened and patched into chunks of size 1×4096 and reshaped to 4×32 ×32, similar to the dose maps. 6) To enhance subtle voxel Hounsfield Unit (HU) differences in the H&N region, we non-linearly mapped the voxel HU values to a range of 0 to 1, with a particular emphasis on the range from −200 to 300 HU, where most soft tissues and tumors are likely to fall. The voxels having HU numbers less than - 1000 and greater than 29,000 were considered as outliers and were clipped to −1000 and 29,000, respectively. The details of the correspondence between the original HU number and preprocessed CT values are shown in Table 1.

**Figure 1.**
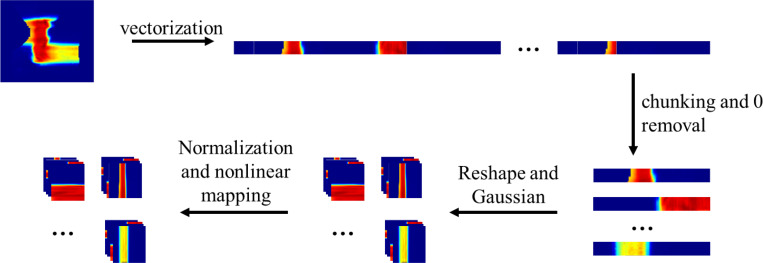
Data preprocessing pipeline. All inputs — including the noisy dose, high statistics dose, and the corresponding CT images — were preprocessed consistently. Zero-value regions were identified based on the noisy dose; once these volumes with zero-value dose were excluded, the corresponding CT volumes were removed as well. A Gaussian filter was applied exclusively to the noisy dose. The dose was normalized using the log1p function computing the natural logarithm of (1+x), where x is the raw value. The nonlinear mapping applied to the voxel HU numbers is detailed in Table 1. During the inference stage, the final denoised dose was reconstructed by spatial concatenation of all the denoised chunks.

7) Both dose maps and CT image matrices were transposed and steps one through five were repeated for better superior-inferior direction continuity and increasing the diversity of the training samples. The data preprocessing pipeline is shown in Figure 1. During the inference stage, the final denoised dose was reconstructed by reversely mapping to the original value and spatially concatenating all denoised dose chunks.

Among 90 patients, 10 patients were randomly reserved for testing and the remaining 80 were used for training. The structures associated with the CT images were not utilized in the training. With the proposed data preprocessing pipeline, the training set included 124,493 triplets consisting of noisy dose, high statistics dose and the corresponding CT image. For each triplet, the chunks of noisy dose maps and CT images served as inputs for the network, while the high statistics dose maps were considered ground truth.

### Diffusion Transformer-based Dose Denoising Framework

**Figure 2.**
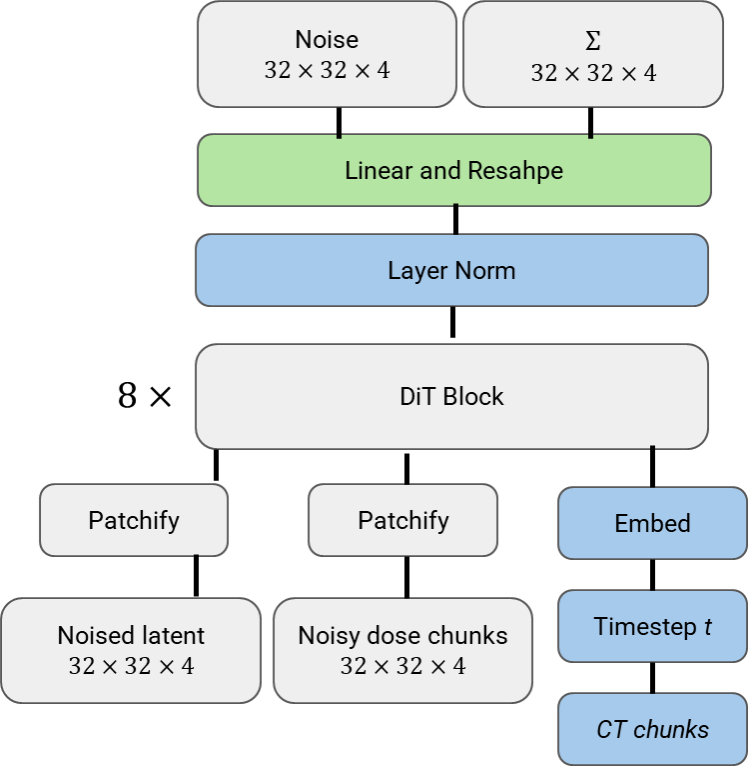
Model architecture. The noisy dose (low-statistic dose map) together with the diffusion-based noised latent embeddings derived from the high-statistic dose map served as the input for the model, and the corresponding CT images were utilized as the conditions. Across all experiments, the proposed framework employed 8 DiT blocks. Standard transformer blocks with adaptive layer normalization were adopted due to their superior performance. For further details on the transformer variants, please refer to Peebles et al.^46^.

The diffusion transformer (DiT)^46^, targeted for high-fidelity, high-resolution natural image generation, was employed as the backbone in our framework (Figure 2). Before elaborating the details of dose denoising framework, we briefly reviewed some basic concepts about denoising diffusion probabilistic models (DDPMs)^47–49^. Gaussian diffusion models have two main steps— forward process and reverse process (denoising). In the forward process, Gaussian noise is gradually added to the real data $x_{0}$ following equation: $q\left(x_{t}|x_{0}\right)=N\left(x_{t};\sqrt{{\bar{a}}_{t}}x_{0},1-{\bar{a}}_{t}I\right)$, where $a_{t}=1-\beta_{t}$ and ${\bar{a}}_{t}=\prod_{s=1}^{t}a_{s}$ with $\beta_{t}$ being the variance schedule controlling how much noise is added at each step. With the reparameterization trick, $x_{t}$ can be sampled as $x_{t}=N\left(x_{t};\sqrt{{\bar{a}}_{t}}x_{0}+\sqrt{1-{\bar{a}}_{t}}\epsilon_{t}\right)$, where $\epsilon_{t}∼N(0,I)$. The noise is added via multiple steps until the original image is completely destroyed, leaving nearly pure Gaussian noise. This step is similar to a Markov chain process, where information is progressively lost. In the reverse process, the diffusion models are trained to invert forward process corruptions, reconstructing original data or generating entirely new samples from noise step by step:$p_{\theta }\left(x_{t-1}|x_{t}\right)=N\left(\mu_{\theta }\left(x_{t}\right),\sum_{\theta }\left(x_{t}\right)\right)$, where neural networks aims at predicting the statistics of $p_{\theta }$. The reverse process model is trained with the variational lower bound^50^ of the log-likelihood of $x_{0}$. Since direct computation is intractable, the objective is to maximize the evidence lower bound (ELBO):

(1) $$log\;p_{\theta }\left(x_{0}\right)\geq {\text{E}}_{\text{q}}\left[\sum_{t=1}^{\text{T}}-D_{KL}\left(q\left(x_{t}|x_{t-1}\right)\|p_{\theta }\left(x_{t}|x_{t+1}\right)\right)+log\;p_{\theta }\left(x_{0}|x_{1}\right)\right],$$

where $D_{KL}$ ensures the learned reverse process $p_{\theta }\left(x_{t}|x_{t+1}\right)$ matches the true noise distribution, and $log\;p_{\theta }\left(x_{0}|x_{1}\right)$ ensures proper reconstruction of the original data. Since both q and $p_{\theta }$ are Gaussian, $D_{KL}$ can be evaluated with the mean and covariance of the two distributions. Thus, with some reparameterization tricks, the training can be simplified to let the network predict the noise ϵ, leading the loss to be simplified as:

(2) $$L_{simple}={\text{E}}_{x_{0},\epsilon ,t}\left[{\left\|\epsilon -\epsilon_{\theta }\left(x_{t},t\right)\right\|}^{2}\right]$$

where $\epsilon_{\theta }\left(x_{t},t\right)$ is the network’s prediction of the noise at time t. Once $p_{\theta }$ is trained, new images can be sampled by initializing $x_{tmax}∼N(0,I)$ and sampling $x_{t-1}∼p_{\theta }\left(x_{t-1}|x_{t}\right)$.

In DiT, an additional off-the-shelf pre-trained variational autoencoder (VAE) model^50^ from Stable Diffusion^49^ was adopted to achieve faster training/inference while preserving image quality and structure efficiently via casting the raw images into a lower-dimensional latent space and operating in the well-structured latent space. UNet was replaced by transformer blocks in DiT, making it easier to capture long-term dependencies and achieve superior scalability and adaptability.

To better accommodate the DiT model to the ultra-sparse and ill-distributed dose data and fully utilize prior knowledge from noisy dose and associated CT images, the following improvements regarding the model architecture were made: 1) The encoder and decoder of the pre-trained VAE model were removed because they were designed for natural images, which differ fundamentally from the dose data. As a result, the well-structured latent space was unsuitable for representing the dose data. 2) To fully leverage the prior knowledge from the noisy dose and CT images, we formed the inverse problem as a Markov chain conditioned on the noisy dose map $x_{noise}$ and CT image $y_{ct}$ and the high statistics dose map, $x_{0}$_,_ can be achieved by $p_{\theta }\left(x_{t-1}|x_{t},x_{noise},y_{ct}\right)$. The ELBO in Equation (1) is updated to:

(3) $$log\;p_{\theta }\left(x_{0}|y\right)\geq {\text{E}}_{\text{q}\left(x_{1:T}|x_{0},x_{noise},y_{ct}\right)}\left[\sum_{t=1}^{\text{T}}-D_{KL}\left(q\left(x_{t}|x_{t-1},x_{noise},y_{ct}\right)\|p_{\theta }\left(x_{t}|x_{t+1},x_{noise},y_{ct}\right)\right)\;+log\;p_{\theta }\left(x_{0}|x_{1},x_{noise},y_{ct}\right)\right],$$

and similarly, ELBO can be rewritten in terms of denoising score matching:

(4) $$L_{simple}={\text{E}}_{x_{0},\epsilon ,t}\left[{\left\|\epsilon -\epsilon_{\theta }\left(x_{t},t,x_{noise},y_{ct}\right)\right\|}^{2}\right]$$

where the model $\epsilon_{\theta }\left(x_{t},t,x_{noise},y_{ct}\right)$ predicts the noise conditioned on both the noisy sample $x_{t}$ and the prior knowledge noisy dose map $x_{noise}$ and CT images $y_{ct}$. 3) The number of DiT blocks was limited to 8 to avoid gradient vanishing and potential overfitting performance considering the relatively limited dose data. 4) Binary CT masks $M_{ct}$ were generated on the fly such that all voxels corresponding to non-zero dose, along with their five surrounding voxels, are assigned a value of 1, while all remaining elements are set to 0. 5) Masked CT images $y_{ct}=M_{ct}*CT_{ori}$ were then embedded and input as the conditions for better focusing on the region of interests (ROI), *i.e,* non-zero dose region.

The proposed framework was trained with Equation (4) as one of the objective functions. As the pretrained VAE was not included in our framework, the reconstruction loss and KL-divergence loss that constrained the VAE network were dropped from our framework as well. Besides, a weighted mean absolute error (wMAE) loss was adopted for emphasizing the voxels with relatively high dose, which usually corresponds to the critical voxels in the CTV region. Moreover, we introduced a residual loss that specifically targeted voxels with the top 10% and bottom 10% dose values. This residual loss penalizes the deviation between the residuals (differences from the noisy dose) of the denoised dose and the ground truth dose. Specifically, it measures the mean absolute difference between the absolute residuals of the prediction and ground truth with respect to noisy dose, ensuring improved accuracy in the pre-Bragg region and distal-fall off region, and smoothness in the spread-out Bragg peak (SOBP) region. Thus, the overall objective function for training the proposed framework is:

(5) $$\begin{array}{l} L=L_{simple}+L_{WMAE}+L_{resi}\\ ={\text{E}}_{x_{0},\epsilon ,t}\left[{\left\|\epsilon -\epsilon_{\theta }\left(x_{t},t,x_{noise},y_{ct}\right)\right\|}^{2}\right]+{\text{E}}_{x_{0},\epsilon ,t}\left[x_{0}\times {\left\|\left(x_{0}-{\hat{x}}_{0}\right)\right\|}^{2}\right.\\ +{\text{E}}_{x_{0},\epsilon ,t}\left[\left(M_{x_{0}}\times \|\|{\hat{x}}_{0}-x_{noise}\|-\|x_{0}-x_{noise}\|\|\right.\right] \end{array}$$

where the predicted dose map ${\hat{x}}_{0}=\frac{x_{t}-\sqrt{1-{\bar{a}}_{t}}\epsilon_{\theta }\left(x_{t},t,x_{noise,}y_{ct}\right)}{\sqrt{{\bar{a}}_{t}}}$ and $M_{x_{0}}$ represented the mask of top 10% and bottom 10% dose values.

### Model evaluation and statistical analysis

To quantitively assess the discrepancies between the predicted dose map ${\hat{x}}_{0}$ and ground truth dose map $x_{0}$, we measured absolute differences using mean absolute error (MAE) between the predicted and ground truth dose maps across the 10 H&N patients reserved for test. Dose volume histogram (DVH) indices were calculated to visualize differences between the predicted and ground-truth dose maps. Besides, with special emphasis on treatment plan quality, we also evaluated CTV D_98%_—the minimum dose normalized by the prescription dose to cover 98% of the target—and CTV D_2%_ for evaluating target dose conformality and homogeneity, respectively. Clinically relevant DVH indices of OARs were calculated and reported for patients of the four distinct disease sites included in this study. 3D Gamma passing rate was calculated to evaluate the dose difference between the denoised and ground truth doses. The t-test was utilized to compare all evaluation metrics and p-values below 0.05 were deemed statistically significant. Furthermore, to quantitively test the generalizability of the framework, we repeated the evaluations and analysis on another 30 patients including 10 with prostate cancer, 10 with breast cancer, and 10 with lung cancer.

## Results

### Dose Distribution Comparison

**Figure 3.**
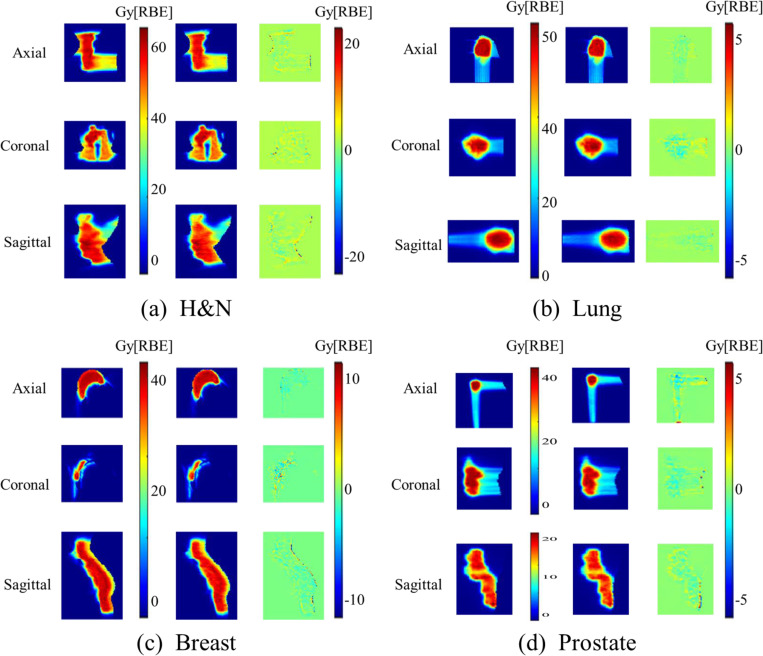
Comparison between the ground truth (planned) dose distributions and the denoised dose predictions across three orthogonal planes: axial, coronal, and sagittal. Four representative clinical cases are shown: (a) H&N, (b) lung, (c) breast, and (d) prostate. The first column of each panel shows the ground truth dose, the second column of each panel shows the predicted dose, and the third column shows the difference between the ground truth and predicted doses. The visual comparison highlights the spatial agreement between the reference and predicted doses, demonstrating the model’s performance across different anatomical sites and imaging orientations.

We evaluated the dose distribution quality by computing the MAE between the reference (clean) and denoised dose distributions, reported in both Gray (Gy[RBE]) and percentage error relative to the maximum dose. For H&N cases, our framework achieved strong performance with an MAE of . 195 ± .112 Gy[RBE] and . 285 ± .145% (p<.05) in the whole BODY; . 124 ± .225 Gy[RBE] and . 282 ± .123 % (p<.05) for CTV and . 086 ± .174 Gy[RBE] and . 165 ± .251% (p < .05) for OARs. Figure 3 (a) illustrates the planned and denoised dose distributions for a representative H&N case displaying the dose distributions in the axial, coronal, and sagittal planes at three randomly selected positions. As illustrated in Figure 3 (a), the deviations between the denoised and ground truth doses were minimal. To further assess and validate the model generalization across disease sites not included in training, we also evaluated it on 10 lung cases, 10 breast cases, and 10 prostate cases respectively. Despite being trained on H&N data only, the model maintained promising performance, with the MAE value within the whole BODY consistently below 0.4 Gy[RBE]. Specifically,

- Lung cases: . 12 ± .054 Gy[RBE] and 0.20 ± .09 % (p<.05) for the whole BODY; 1.08 ± 2.03 Gy[RBE] and 1.62±2.21 % (p<.05) for CTV and . 36 ± .22 Gy[RBE] and . 53 ± .61 % (p<.05) for OARs.
- Breast cases: . 17 ± .09 Gy[RBE] and . 35 ± .16 % (p<.05) for the whole BODY; 1.06 ± .58 Gy[RBE] and 2.32 ± .72 % (p<.05) for CTV and . 13 ± .46 Gy[RBE] and . 52 ± .24 % (p<.05) for OARs.
- Prostate cases: . 37 ± .37 Gy[RBE] and . 62 ± .67 % (p<.005) for the whole BODY. 1.02 ± 1.52 Gy[RBE] and 2.44 ± 1.76 % (p<.05) for CTV and . 02 ± .02 Gy[RBE] and 1.72 ± .09 % (p<.05) for OARs.

Figure 3 (b)–(d) shows presentative results for lung, breast and prostate cases respectively, displaying the dose distributions in three orthogonal planes at three randomly selected positions. These results highlight the model’s ability to recover the ground truth dose distributions from noisy dose, even in diseases sites that the model was not explicitly trained on. The negligible difference between the denoised and ground truth dose across different disease sites, demonstrated strong cross-site adaptability and robustness of the proposed model.

### DVH Comparison

**Figure 4.**
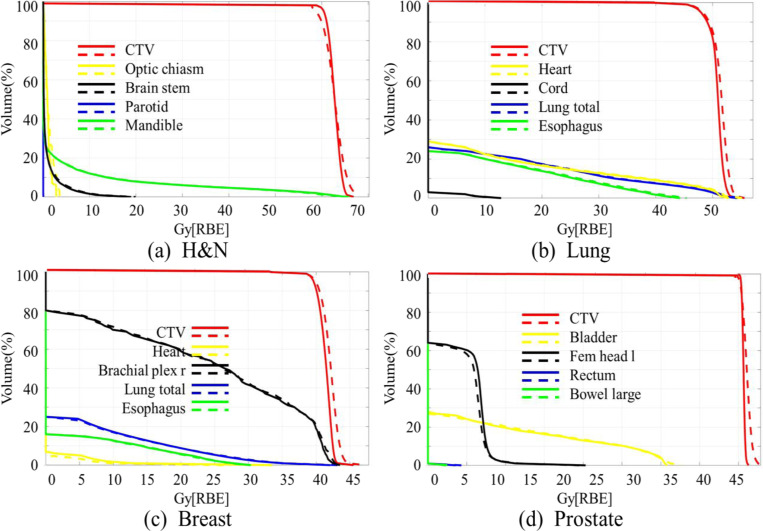
Comparison of DVHs for CTV and the selected OARs between the ground truth dose (solid lines) and the denoised dose (dashed lines) for a typical H&N, lung, breast and prostate case, respectively.

We calculated the DVH indices for the CTV and OARs based on the denoised dose and ground truth doses respectively from 4 representative cases with the results summarized in Figure. 4. Across all disease sites, the differences in DVH metrics were minimal, indicating that the denoised dose maintained strong agreement with the reference dose in clinically relevant regions. Specifically, for the CTV, the D_98%_ (dose received by 98% of the volume) was . 93 ± .25 Gy[RBE], while the D_2%_ (dose received by the hottest 2% of the volume) was . 235 ± .516 Gy[RBE] when comparing the denoised to ground truth doses. These values indicate that the target dose conformality and homogeneity were well-preserved following denoising.

For OARs, differences in DVH indices between the denoised and ground truth doses were visually negligible, suggesting that the proposed framework effectively maintains critical DVH index constraints in OARs.

### 3D Gamma Passing Rate Evaluation

**Figure. 5.**
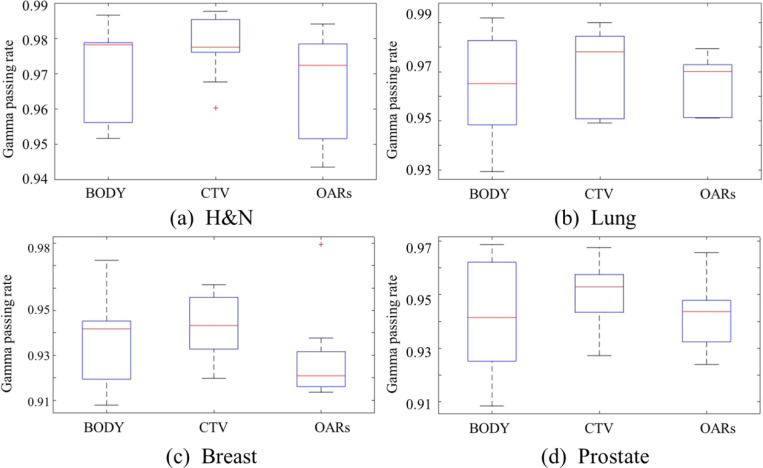
Boxplots showing the 3D Gamma passing rates (using 3%/2mm criteria) for BODY, CTV, and OARs across four disease sites: H&N, lung, breast, and prostate. The central line in each box indicates the median, with the box edges representing the interquartile range (IQR) and whiskers extending to the most extreme data points.

To further quantitatively evaluate the accuracy of the dose distributions, 3D Gamma passing rates were calculated for CTV, OARs, and BODY for all the testing cases. On average, the 3D Gamma passing rate was above 92% with criterion of 3%/2mm regardless of the structures in the whole BODY across different disease cites. To be specific, for the H&N cases, we achieved 95.93% ± 1.23% for the whole BODY, 97.98% ± 0.53% for CTV and 97.61% ± 1.13% for OARs. For the lung cases, the 3D Gamma passing rate was 97.48% ± 1.03% for the whole BODY, 95.67% ± 1.32% for CTV and 94.96% ± 2.12% for OARs. For the breast cases, the 3D Gamma passing rate was 93.53% ± 1.28% for the whole BODY, 92.78% ± 1.73% for CTV and 94.40% ± 2.12% for OARs. For the prostate cases, the 3D Gamma passing rate was 94.98% ± 1.30% for the whole BODY, 95.41% ± 0.98% for CTV and 93.04% ± 1.43% for OARs. Figure 5 showed the boxplot regarding the 3D Gamma passing rate. The central line in each box indicates the median, with the box edges representing the interquartile range (IQR) and whiskers extending to the most extreme data points.

## Discussion

In this study, we proposed a novel universal deep learning-based framework for dose denoising, leveraging a DiT backbone to achieve robust performance across various disease sites. In contrast to many existing deep learning-based dose denoising models—which often rely on tightly controlled datasets with constraints such as fixed image dimensions, specific disease sites or subgroups, uniform beam configurations, or consistent treatment planning strategies, our framework is intentionally designed to relax these restrictions. This inherent flexibility in training data requirements enhances its generalizability, making it particularly well-suited for clinical settings, where such uniformity is difficult to ensure.

### A key component supporting this generalizability is our universal data preprocessing strategy.

Deep learning models, by nature, are data-driven and task-focused; they operate by learning mappings between inputs and outputs, without the need to interpret the underlying physical mechanisms of the data. Leveraging this principle, we devised a data formatting method that reconfigures heterogeneous datasets into standardized patches while preserving the spatial and contextual relationships between input data. This approach allows the model to be trained on a diverse dataset and still generalize effectively to independent cases that may differ significantly from the training data.

### Articulately processed conditional data and model architecture boosted the model performance.

Specifically, we normalized the HU values of the CT images to a fixed range, with particular attention given to the HU values in the range of −300 to 200. This range, which is the normal values for soft tissue and tumors, was given high priorities to steer the model to focus on more clinically relevant areas. This normalization step not only improved training stability but also ensured the model learned meaningful spatial correlations.

Finally, recognizing the practical limitations in data availability in real-world clinical scenarios and the huge difference between medical images and natural images, we opted for a lightweight version of the DiT architecture. By reducing the number of transformer blocks compared to the original design, we minimized the risk of overfitting while maintaining strong denoising performance. Moreover, lightweighted framework also requires “light” demands of the computing resources, thus enhancing the framework’s accessibility and scalability across diverse clinical settings. This careful balancing of model complexity, training data requirements, and computing resource needs underscores the practicality and adaptability of our proposed framework in real-world clinical scenarios.

Nevertheless, in real-world clinical scenarios, achieving a balance between model accuracy and generalizability remains critical. When encountering previously unseen patient data, it is inherently challenging for a universal model to outperform specialized models that have been fine-tuned for a particular data domain and specific task. For example, although our framework achieved consistently good 3D Gamma passing rates overall in the whole BODY, there were isolated instances where the 3D Gamma passing rates fell below 95% , particularly in more complex cases. Consequently, for applications that demand high precision, it is advisable to fine-tune the framework using a small, task-specific dataset or to preprocess the data to better align with the data distribution used during the model’s training stage. Such strategies will help improve both reliability and adaptability in scenarios where fine-grained accuracy is demanding.

Additionally, we observed that the 3D Gamma passing rate for the CTV was not consistently higher than that for the whole BODY, despite CTV being of greater clinical importance. This can be attributed to two factors: 1) The inherent characteristics of the framework. We utilized DiT backbone, which is great at capturing global contextual features but does not prioritize specific anatomical regions; 2) The absence of task-specific guidance. We did not introduce any auxiliary information to explicitly emphasize CTVs or OARs, thus leading the model to treat all voxels with equal importance. Therefore, for the potential future clinical deployment, if precision on specific targets (e.g., CTVs or OARs) is essential, it is recommended to incorporate more prior knowledge during training or inference, such as structure contours or patient-specific information. This will help the model weigh more attention toward more clinically relevant regions and improve its effectiveness in specialized tasks.

## Conclusions

In this study, we introduced a novel universal dose denoising framework based on the DiT architecture, designed to effectively reconstruct high statistics dose distributions from noisy ones from Monte Carlo simulations in proton therapy. A notable advantage of this approach is its robustness and generalizability. Although it was trained using data from only H&N cancer patients, it can accurately and robustly denoise noisy dose maps across different disease sites including breast, prostate and lung without any fine-tuning or re-training, making the framework practical for implementation in routine clinics. Importantly, the framework does not rely on strict assumptions or constraints regarding the data input. It accommodates a wide range of clinical variations including differences in image dimensions, prescription doses, and beam configurations. This flexibility distinguishes from many existing deep learning-based dose denoising models that often require extensive input data standardization or site-specific data selection. As a result, the proposed method offers a more practical, versatile, and scalable solution for dose denoising in routine clinics in proton therapy, with minimal efforts for data preprocessing or postprocessing.

## Figures and Tables

**Table 1:** Nonlinear mapping of CT voxel Hounsfield Unit (HU) values normalized to a range of 0 to 1, with emphasis on the clinically relevant HU range of −200 to 300. The table illustrates the transformation applied to the original voxel HU values using a predefined mapping function.

Original CT value (HU)	Normalized value (0–1)	Mapping Equation
< −200	0~0.1	$\frac{CT-(-1000)}{-200-(-1000)}\times 0.1$
≥ −200 & < 300	0.1~0.7	$0.1+\frac{CT-(-200)}{300-(-200)}\times (0.7-0.1)$
≥ 300 & < 3000	0.7~0.85	$0.7+\frac{CT-300}{3000-300}\times (0.85-0.7)$
≥ 3000 & < 29000	0.85~1	$0.85+\frac{\log(CT-3000+1)}{\log(29000-3000+1)}\times (1-0.85)$

## Data Availability

Research data are stored in an institutional repository and will be shared upon request to the corresponding author.
